# GPC-YOLO: An Improved Lightweight YOLOv8n Network for the Detection of Tomato Maturity in Unstructured Natural Environments

**DOI:** 10.3390/s25051502

**Published:** 2025-02-28

**Authors:** Yaolin Dong, Jinwei Qiao, Na Liu, Yunze He, Shuzan Li, Xucai Hu, Chengyan Yu, Chengyu Zhang

**Affiliations:** 1 School of Mechanical and Automotive Engineering, Qilu University of Technology (Shandong Academy of Sciences), Jinan 250353, China; 10431220003@stu.qlu.edu.cn (Y.D.); insistein@163.com (N.L.); 10431220034@stu.qlu.edu.cn (S.L.); a97868@163.com (X.H.); 15106981578@163.com (C.Y.); 15692371440@163.com (C.Z.); 2 Shandong Institute of Mechanical Design and Research, Jinan 250353, China; 3 College of Electrical and Information Engineering, Hunan University, Changsha 410082, China; hejicker@163.com; 4 National Engineering Research Center of Robot Visual Perception and Control Technology, Changsha 410082, China

**Keywords:** YOLOv8, lightweight, tomato, maturity, C2f-PC

## Abstract

Effective fruit identification and maturity detection are important for harvesting and managing tomatoes. Current deep learning detection algorithms typically demand significant computational resources and memory. Detecting severely stacked and obscured tomatoes in unstructured natural environments is challenging because of target stacking, target occlusion, natural illumination, and background noise. The proposed method involves a new lightweight model called GPC-YOLO based on YOLOv8n for tomato identification and maturity detection. This study proposes a C2f-PC module based on partial convolution (PConv) for less computation, which replaced the original C2f feature extraction module of YOLOv8n. The regular convolution was replaced with the lightweight Grouped Spatial Convolution (GSConv) by downsampling to reduce the computational burden. The neck network was replaced with the convolutional neural network-based cross-scale feature fusion (CCFF) module to enhance the adaptability of the model to scale changes and to detect many small-scaled objects. Additionally, the integration of the simple attention mechanism (SimAM) and efficient intersection over union (EIoU) loss were implemented to further enhance the detection accuracy by leveraging these lightweight improvements. The GPC-YOLO model was trained and validated on a dataset of 1249 mobile phone images of tomatoes. Compared to the original YOLOv8n, GPC-YOLO achieved high-performance metrics, e.g., reducing the parameter number to 1.2 M (by 59.9%), compressing the model size to 2.7 M (by 57.1%), decreasing the floating point of operations to 4.5 G (by 45.1%), and improving the accuracy to 98.7% (by 0.3%), with a detection speed of 201 FPS. This study showed that GPC-YOLO could effectively identify tomato fruit and detect fruit maturity in unstructured natural environments. The model has immense potential for tomato ripeness detection and automated picking applications.

## 1. Introduction

Tomatoes are one of the most cultivated fruits worldwide, consumed raw or cooked. Rich in lycopene, tomatoes possess strong antioxidant properties that can lower blood pressure and reduce the risk of heart disease [1]. However, unripe tomatoes contain tomatine, which is toxic if consumed in excess [2]. Tomatoes grow in clusters, but tomatoes on the same cluster do not mature simultaneously. If the ripe tomatoes are not picked in time, the ripe tomatoes rot over time and affect the development of the immature tomatoes. In addition, in greenhouse environments, tomatoes mature rapidly, limiting the regular harvesting of ripe fruits every few days. The lack of selective ability and the low intelligence level of mechanized picking result in approximately 20% of unripe tomatoes mixed in during the mechanical harvesting process [2,3]. Therefore, achieving accurate fruit picking requires a correct identification of tomatoes at different maturity stages. With the aging population and increasing labor costs, robotic automated picking has played a vital role in agriculture [4].

The application of harvesting robots in unstructured agricultural environments requires essential technologies, including fruit visual recognition [5], real-time target localization [6], as well as path planning methods [7,8]. Among these, fruit visual recognition technology determines if the harvesting robot can accurately obtain target information. In recent years, deep learning-based target detection methods have rapidly advanced in the field of agricultural automation, particularly convolutional neural networks (CNNs), which can achieve detection accuracies and speeds comparable to the work of human beings in some fruit detection and automatic harvesting fields [9]. This study focuses on recognizing tomatoes in greenhouses and applied deep learning algorithms to detect and classify tomatoes into different maturity stages.

In fruit detection, image processing techniques can be classified into traditional and deep learning-based image processing. The former is sensitive to environmental changes, which can lead to inaccurate processing results in environments with unstable lighting conditions. At the same time, they cannot fully capture the complex morphology and surface texture of the fruit, affecting the classification and recognition accuracy. Traditional image processing technology has limitations in scalability and is difficult to adapt to varieties of fruits and different agricultural environments. Each new application or environmental change requires redesigning and modifying the algorithm [10]. These shortcomings have prompted researchers to gradually turn to deep learning technology. In fruit-picking applications, digital image processing technology based on deep learning has many advantages over traditional methods. Deep learning models (such as CNNs) can automatically learn complex features in images, have high classification and recognition accuracy, have strong robustness, and cope with various lighting conditions, background complexity, and changes in different perspectives [11]. Through large amounts of data training, deep learning models can automatically adjust the parameters to adapt to different environments and conditions without manual parameter adjustment. Deep learning-based fruit detection algorithms are primarily categorized into two-stage detection methods [12] and single-stage detection methods [13]. The two-stage target detection framework separates the target localization and target classification tasks, such as Faster R-CNN [14] and Mask R-CNN [15]. The single-stage target detection framework uses a deep convolutional neural network to perform localization and classification simultaneously without separating the two steps, such as SSD [16], YOLO [17,18], and RetinaNet [19]. Among these methods, the YOLO series of target detection networks stand out for their rapid detection speed. However, YOLO is a multi-category target detection algorithm. Only a few categories are involved in the fruit maturity detection task. Therefore, many network parameters are redundant, including the macrostructure and microstructure. This causes overfitting, leading to an excessive model size [20]. The model size should be minimized to improve network efficiency while maintaining model accuracy. In recent years, researchers have focused on developing lightweight fruit detection algorithms, particularly emphasizing improvements derived from the YOLO framework. Liu et al. [21] introduced a lightweight apple detection algorithm, Faster-YOLO-AP, which was further refined into a smaller-scale network, YOLOv8pico (YOLOv8p). To reduce computational complexity, they proposed a less resource-intensive Partial Depth Convolution (PDWConv), which was used to construct the PDWFasterNet module. For bounding box regression, EIoU loss was introduced, and Depthwise Separable Convolution (DWSConv) replaced standard convolution (SC) to enhance downsampling efficiency. This optimization led to a reduction in floating point operations and parameters to 2.29 G and 0.66 M, respectively, and the model achieved an impressive mAP50:95 of 84.12%. Chen et al. [22] introduced YOLOv8-GP, a method based on key point detection for the synchronized detection of grapes and picking points. This enhanced grape detector was developed by integrating several advanced techniques, including the FasterNetBlock, EMA (Exponential Moving Average), and BiFPN (Bidirectional Feature Pyramid Network), into the YOLOv8n-Pose architecture. Experimental results showed that YOLOv8-GP significantly boosted grape bunch recognition accuracy, increasing it from 86.4% to 89.7%. In addition, the computational complexity was reduced significantly, with GFLOPs (one billion FLOPs per second) reduced from 8.4 G to 6.1 G and the parameters reduced from 3.08 M to 1.61 M, showing a 27.38% and 47.73% reduction, respectively.

Researchers have also performed lightweight optimization on the YOLO algorithm for fruit maturity detection. Zeng et al. [23] introduced a lightweight tomato detection algorithm. The backbone of YOLOv5 was reconstructed using the backbone module of MobileNetV3. The neck was channel-pruned to reduce its size, and a genetic algorithm was employed to optimize hyperparameters, enhancing detection accuracy. As a result, the parameters and FLOPs were compressed by 78% and 84.15%, respectively, while the model achieved a remarkable mAP of 0.969. Additionally, the detection speed on a CPU platform improved to 42.5 ms, a 64.88% increase in performance. Tamrakar et al. [24] introduced a lightweight YOLOv5s-CGhostnet for detecting and counting strawberry maturity levels. CBS and C3 were replaced with Ghost modules, supplemented by convolutional block attention module (CBAM) and SIoU box loss functions. The YOLOv5s-CGhostnet model achieved 91.7% mAP50, with a model size of 5 MB, requiring 9.8 GFLOPs for computation, and delivering an inference time of 5 ms. Xiao et al. [25] introduced an enhanced version of the YOLOv5 algorithm for detecting blueberry fruit maturity by incorporating the ShuffleNet module to create a lightweight deep CNN. They also added the CBAM to boost feature fusion capabilities in the lightweight CNN. The detection recall and mAP50 were 92.0% and 91.5%, respectively. The average detection speed was 67.1 FPS. The improved YOLOv5 algorithm had a model size of 5.65 MB, 2.85 M network parameters, and 5.6 GFLOPs.

In addition to these advancements, numerous studies have specifically focused on tomato maturity detection. Moreira et al. [26] proposed a method combining deep learning (YOLOv4 and SSD MobileNet v2) with an HSV color space model for tomato detection and classification, achieving an F1-Score of 85.81% for detection and a Macro F1-Score of 74.16% for classification. Su et al. [27] developed the SE-YOLOv3-MobileNetV1 network, which improved tomato maturity classification accuracy to 97.5% and significantly reduced detection time. Li et al. [28] introduced the YOLOv5s-tomato model, which achieved an mAP of 97.42% for tomato maturity recognition in greenhouses. Li et al. [29] also proposed the MHSA-YOLOv8 model, which enhanced tomato maturity detection and counting with an mAP50 of 0.864. Wang et al. [30] proposed a lightweight YOLOv5n-based algorithm for cherry tomato maturity detection, achieving an mAP of 95.2% and a detection speed of 5.3 ms. Wu et al. [31] introduced the MTS-YOLO model, which achieved an mAP@0.5 of 92.0% for tomato maturity and stem detection. Wei et al. [32] developed the GFS-YOLO11 model, which improved multi-variety tomato maturity detection with an mAP50 increase of 6.2%. Wang et al. [33] proposed an improved YOLOv8 algorithm for tomato maturity detection in complex scenarios, achieving an mAP of 86.9% and a recall of 82.0%. Gao et al. [34] introduced the YOLOv8n-CA model, which achieved an mAP of 97.3% for tomato maturity recognition.

Many researchers have achieved lightweight optimization and fruit maturity detection using the improved YOLO model. The agricultural environment background of tomato fruit is relatively complex during ripening. Affected by unstructured factors, such as overlapping and occlusion of tomato fruits and changes in lighting conditions, it is relatively difficult to identify severely stacked tomato fruit targets. In addition, current deep learning detection algorithms typically demand substantial computing resources and memory. This study proposes a model for detecting tomato fruit based on YOLOv8n. The improved model can be used in unstructured natural environments to detect tomato fruit and their maturity stages, supporting subsequent automated tomato picking.

## 2. Materials and Method

### 2.1. Dataset Preparation

#### 2.1.1. Image Acquisition

**Figure 1:**
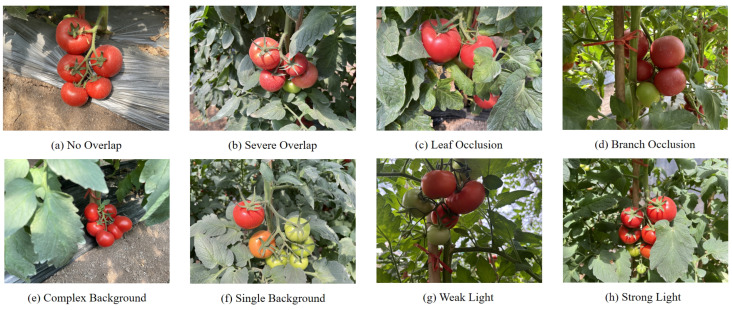
Images of tomatoes under different conditions.

The dataset of tomato was sourced from Shandong Anxin Seed Limited by Share Ltd. in Jinan, Shandong Province, China. Image acquisition was performed using an iPhone camera and a Huawei camera. Subjects were positioned at distances of 0.3 to 0.6 m, and the images were taken under natural lighting conditions, considering unstructured environments with varying light intensities, overlapping objects, and occlusions, as shown in Figure 1. The dataset comprised 1249 images.

**Figure 2:**
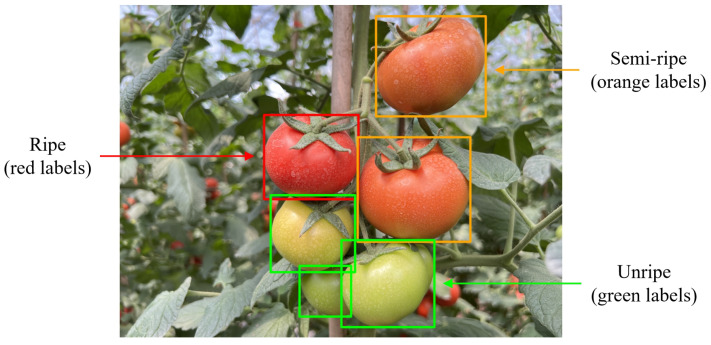
Tomatoes at different maturation stages.

In this section, the process of tomato fruit detection and fruit maturity classification is discussed. The tomatoes were categorized into three distinct maturity stages based on their color transformation during ripening: ripe (denoted by red labels), representing tomatoes that have fully completed color transformation; semi-ripe (denoted by orange labels), indicating tomatoes that have initiated but not fully completed color change; and unripe (denoted by green labels), corresponding to tomatoes that have not yet undergone any color transformation. LabelImg software version 1.8.6 (https://github.com/HumanSignal/labelImg accessed on date 26 February 2025) was used to label the tomatoes in the image. Figure 2 shows tomatoes at different maturation stages.

#### 2.1.2. Image Preprocessing

**Figure 3:**
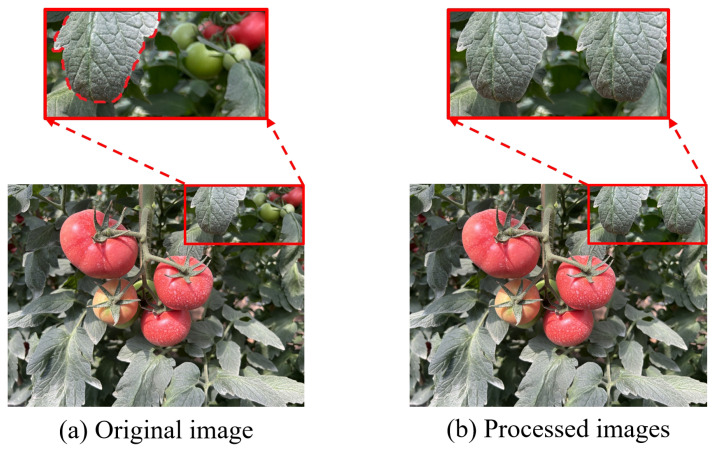
Image processing of tomatoes in the distance.

The aim of this study is to improve the detection model for subsequent applications in tomato-picking robots. Considering the field of view of the camera, the image will inevitably contain tomatoes that are far away from the camera. In the actual picking process, the robot will not pick these distant tomatoes. To eliminate their impact on model training and subsequent picking tasks, Adobe Photoshop CC 2018 software (https://www.adobe.com/products/photoshop.html accessed on date 26 February 2025) was used to cut out the leaves in the image and overlay them on the distant tomatoes, maintaining consistency with the operation of the picking robot (Figure 3). This operation aims to ensure the model’s focus, avoiding interference from tomatoes in the background of the field of view, thereby enhancing the detection accuracy of target tomatoes. Additionally, by ensuring data consistency, it aligns the training data with the picking scenario, preventing the model from learning irrelevant features. Moreover, the practical application demands the simulation of real scenarios, and the retouching overlay can effectively improve the model’s practicality and robustness.

**Figure 4:**
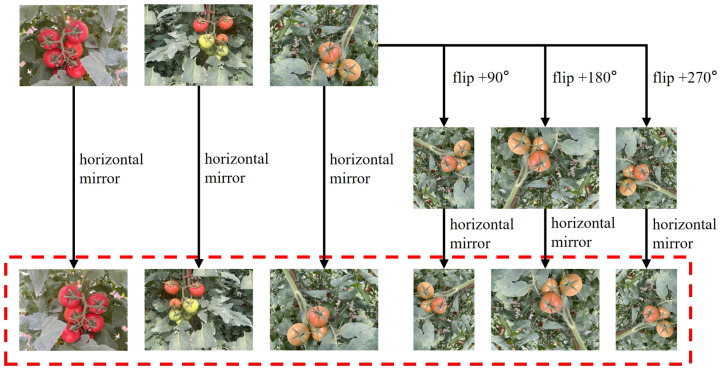
Data augmentation flowchart.

The sample of semi-ripe tomatoes was insufficient because of the imbalance in the proportions of tomatoes at three maturity stages (ripe, semi-ripe, and unripe) in the original data, with the proportions being approximately 12:5:10. Three different rotation angles were used to increase the number of semi-ripe tomato samples and prevent overfitting caused by the limited number of images in the training dataset, i.e., +90°, +180°, and +270°, expanding the number of images containing multiple semi-ripe tomatoes from 304 to 1216. Subsequently, the original and rotated images were flipped horizontally, ultimately expanding the dataset to 4322 images, totaling 19,600 tomato target samples. Figure 4 shows the image enhancement process. The proportions of ripe, semi-ripe, and unripe tomatoes were adjusted to approximately 11:10:10. This effectively alleviates the class imbalance and enhances the dataset.

The dataset was divided into three groups with an 8:1:1 distribution: training, validation, and testing. Specifically, 3457 images were allocated for training, 432 for validation, and 433 for testing.

### 2.2. Model Construction

#### 2.2.1. YOLOv8n Network Structure

**Figure 5:**
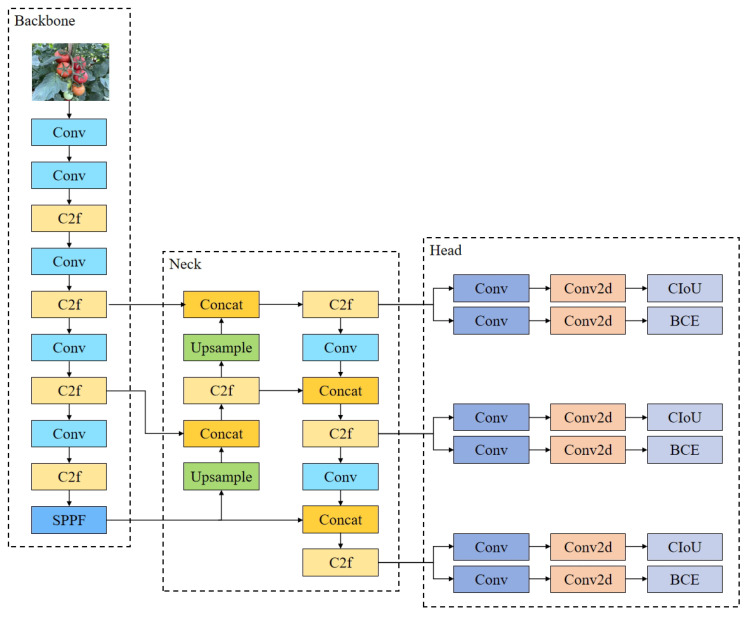
Network architecture of YOLOv8.

The network architecture of YOLOv8 (https://github.com/ultralytics/ultralytics accessed on date 26 February 2025) mainly consists of three parts, as shown in Figure 5: backbone, neck, and head. The backbone network is responsible for feature extraction and forwards the extracted features to the neck. The neck is designed to perform feature fusion and dimension adjustments. In YOLOv8, the PAN-FPN structure is utilized, which helps in integrating feature maps from different layers of the backbone. This combination of features enhances the network’s ability to detect objects at different scales and improves accuracy. The final component of the architecture is the head, which predicts the class and location of objects in the image. Based on the features passed from the neck, the head determines the position and category of the detected objects. Additionally, YOLOv8 comes in multiple versions—YOLOv8n, YOLOv8s, YOLOv8m, YOLOv8l, and YOLOv8x—each progressively larger in terms of complexity and performance. The most lightweight version, YOLOv8n, was selected as the baseline algorithm for this study, specifically for enabling automatic tomato detection on mobile devices, balancing performance and computational efficiency.

#### 2.2.2. GPC-YOLO Network Structure

**Figure 6:**
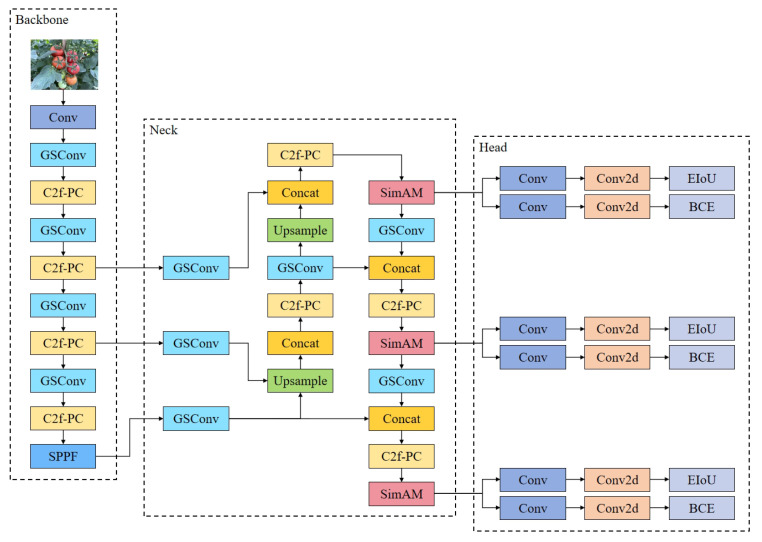
Improved lightweight GPC-YOLO network structure.

Figure 6 shows the lightweight GPC-YOLO tomato detection model. The model consists of three main components: backbone, neck, and prediction head. The improved modules included the following: (1) backbone network, includes the Grouped Spatial Convolution module [35] and C2f-PC module; (2) CNN-based cross-scale feature fusion [36]; (3) simple attention mechanism [37]; and (4) EIoU loss function [38]. By incorporating modules (1) and (2), the Grouped Spatial Convolution and C2f-PC modules greatly minimize computational load and memory consumption, thereby enhancing the network’s efficiency for real-time applications. Meanwhile, modules (3) and (4), incorporating the simple attention mechanism and EIoU loss function, enable the model to effectively detect both tomato fruit and assess their maturity, even in complex and unstructured natural environments. The code of this study is published in https://github.com/YaolinDong/GPC-YOLO accessed on date 27 February 2025.

#### 2.2.3. Grouped Spatial Convolution Module

**Figure 7:**
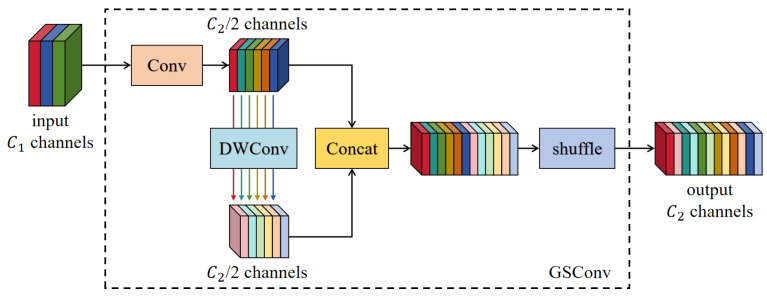
Structure of the GSConv module. The “DWConv” marked in blue means the DSC operation.

The Grouped Spatial Convolution (GSConv) module [35] is a lightweight convolution method, as shown in Figure 7. The GSConv module enhances nonlinear expression capabilities and reduces computational complexity because a channel-sparse convolution computation (DSC) layer and a shuffle operation are added. The SC and GSConv calculations are expressed as Equations (1) and (2), respectively:

(1) $$GFLOPs\left(SC\right)=W\cdot H\cdot K_{1}\cdot K_{2}\cdot C_{1}\cdot C_{2}$$

(2) $$GFLOPs\left(GSConv\right)=W\cdot H\cdot K_{1}\cdot K_{2}\cdot \frac{C_{2}}{2}\cdot \left(C_{1}+1\right)$$

where *W* and *H* denote the width and height of the output feature map. $K_{1}\cdot K_{2}$ is the convolution kernel size, and $C_{1}$ and $C_{2}$ are the input and output channels, respectively. The computational cost of GSConv was about half that of SC (approximately 0.5 + 0.5 $C_{1}$), and as $C_{1}$ increases, the ratio approaches 50%, but it exhibited similar performance to the SC.

#### 2.2.4. C2f-PC Module

**Figure 8:**
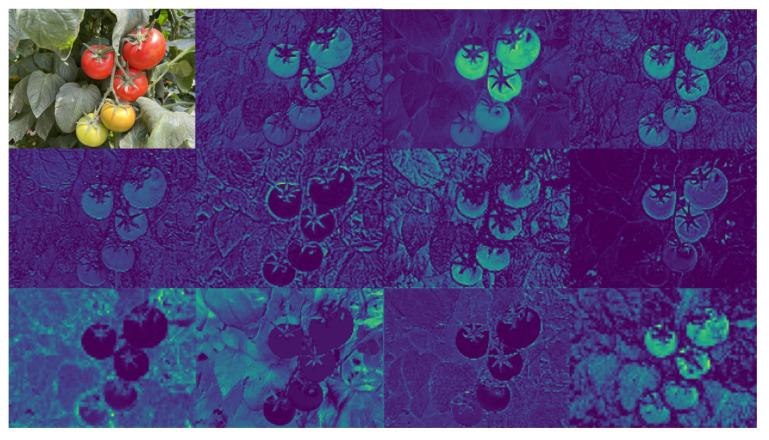
Visualization of the feature map.

**Figure 9:**
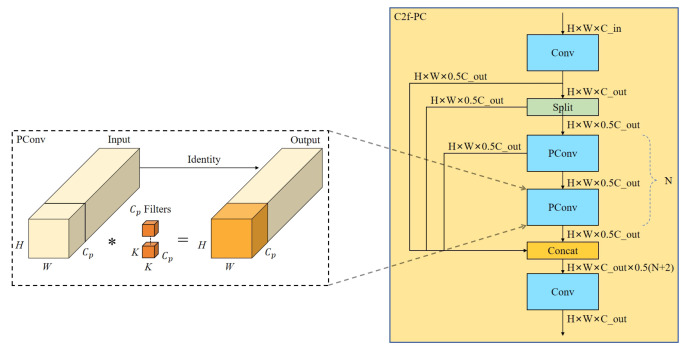
Structure of the C2f-PC module.

In the intermediate stage of the YOLOv8n backbone network, tomato objects were observed across almost all feature map channels, as shown in Figure 8. There is a significant resemblance between the feature maps across different channels. The C2f-PC model was designed by introducing PConv [39] into the C2f module to reduce this computational redundancy. A subset of input channels undergoes an SC through PConv to extract spatial features, while the other channels remain unchanged. The C2f module’s structure is optimized by replacing the SC in the Bottleneck module with PConv, as shown in Figure 9.

The input and output feature maps maintain the same number of channels. The PConv calculation is expressed as Equation (3):

(3) $$GFLOPs\left(PConv\right)=W\times H\times K^{2}\times Cp^{2}$$

Compared to SC, when $\frac{C_{P}}{C_{1}}=\frac{C_{P}}{C_{2}}=\frac{1}{4}$, the calculation amount of PConv is only $\frac{1}{16}$ of SC. This approach significantly reduces the FLOPs required for convolution.

#### 2.2.5. CNN-Based Cross-Scale Feature Fusion

The CNN-based cross-scale feature fusion (CCFF) [36] was introduced in the neck. The objective was to enhance the speed and efficiency of target detection by minimizing network parameters and computational complexity, while simultaneously improving the model’s ability to detect small-scale objects.

CCFF enhances the cross-scale fusion module by integrating multiple fusion blocks into the fusion path. These blocks combine adjacent scale features to generate new feature representations. Information from different levels is combined better, enhancing the overall feature representation capability.

**Figure 10:**
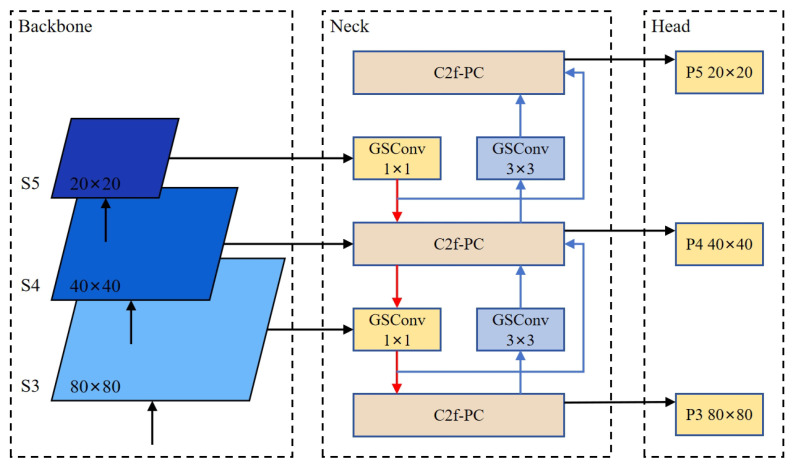
Improved neck part.

At the same time, the fusion block was replaced with the C2f-PC proposed in this study, and Conv was replaced by GSConv to lighten the neck. The network width was reduced to 256 convolutional kernels across all neck modules, significantly lowering the network parameters and improving model speed. Figure 10 presents the improved neck part.

#### 2.2.6. Simple Attention Mechanism

**Figure 11:**
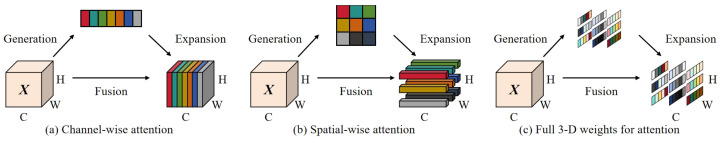
Comparisons of the different attention steps.

Most tomatoes appear as small targets, often overlapping clusters because of the camera distance and angle. Consequently, the feature fusion in the neck network may not be sufficient to accurately capture the multi-dimensionality and correlations among features, which can result in performance degradation when dealing with complex scenarios and small targets. The simple attention mechanism (SimAM) [37] was added to the neck network to address these issues. In contrast to the traditional attention mechanism, SimAM is a 3D attention mechanism that does not introduce additional parameters during the training process. Its rapid calculation speed makes it suitable for training with limited hardware resources, providing a significant advantage over other modules. Figure 11a–c depict the channel attention mechanism, the spatial attention mechanism, and the structure of SimAM.

#### 2.2.7. EIoU Loss

The CIOU loss [40] incorporates three geometric aspects: overlap area, normalized central point distance, and aspect ratio. For a predicted box and a target box, the CIOU loss is formulated as Equations (4)–(7):

(4) $$IoU=\frac{\left|B\cap B^{gt}\right|}{\left|B\cup B^{gt}\right|}$$

(5) $$\alpha =\frac{v}{(1-IoU)+v}$$

(6) $$v=\frac{4}{\pi^{2}}{\left(\arctan\frac{w^{gt}}{h^{gt}}-\arctan\frac{w}{h}\right)}^{2}$$

(7) $$L_{CIoU}=1-IoU+\frac{\rho^{2}\left(b,b^{gt}\right)}{c^{2}}+\alpha v$$

The ground-truth box is denoted as $b^{gt}$, while B represents the predicted box. $B\cap B^{gt}$ and $B\cup B^{gt}$ correspond to their intersection and union. The width-to-height ratio discrepancy is captured by α (a positive trade-off parameter) and *v* (which assesses the aspect ratio consistency). *b* and $b^{gt}$ refer to the central points of *B* and $B^{gt}$, respectively. ρ(·) is the Euclidean distance, and *c* is the diagonal length of the smallest enclosing box that covers both boxes.

The CIOU loss may unreasonably optimize the similarity because it only reflects the difference in aspect ratio. This could hinder the model from effectively reducing the true difference between (w,h) and $\left(w^{gt},h^{gt}\right)$.

These problems were addressed by adopting the EIoU [38] loss function for improvement.

The EIoU loss function is decomposed into three components: IOU loss ($L_{IoU}$), distance loss ($L_{dis}$), and aspect ratio loss ($L_{asp}$). Consequently, EIoU preserves the advantages of CIOU loss while directly reducing the disparities in width and height between the target and anchor boxes, resulting in accelerated convergence and enhanced localization accuracy. Equation (8) defines the definition of EIoU:

(8) $$\begin{array}{cc} L_{EIoU}= & L_{IoU}+L_{dis}+L_{asp}=1-IoU+\frac{\rho^{2}\left(b,b^{gt}\right)}{{\left(w^{c}\right)}^{2}+{\left(h^{c}\right)}^{2}}+\frac{\rho^{2}\left(w,w^{gt}\right)}{{\left(w^{c}\right)}^{2}}+\frac{\rho^{2}\left(h,h^{gt}\right)}{{\left(h^{c}\right)}^{2}} \end{array}$$

where $w^{c}$ and $h^{c}$ denote the width and height, respectively.

### 2.3. Experimental Setting

All experiments for model training and evaluation were conducted on the same hardware, featuring an Ubuntu 20.04 operating system, a 10-core Intel(R) Xeon(R) Platinum 8260M CPU @ 2.30 GHz, and an NVIDIA GeForce RTX 3090 GPU. The training framework was PyTorch 2.0.1, with code implemented in Python 3.10.0, while cuDNN 8.9.0 and CUDA 11.8 were employed for hardware acceleration. The batch size was set to 16, considering the parameters, computational complexity, and memory consumption of networks with different depths and widths. The SGD optimizer was used. The size of the input images was 640 × 640, with momentum, weight decay, initial learning rate, and number of epochs set to 0.937, 0.0005, 0.01, and 300, respectively.

### 2.4. Evaluation Metrics

The performance of the GPC-YOLO model was assessed using precision (P), recall (R), and mean average precision (mAP) as evaluation metrics, which were computed through Equations (9)–(12):

(9) P=TP/(TP+FP)

(10) R=TP/(TP+FN)

(11) $$AP=\int_{0}^{1}P\left(R\right)dR$$

(12) $$mAP=\frac{\sum_{i=1}^{N}AP_{i}}{N}$$

where TP, FP, and FN represent the counts of true positives, false positives, and false negatives, respectively. AP represents the area under the precision–recall (P-R) curve, which serves as a metric for evaluating the performance of the object detection model. mAP is the average of AP across the three tomato detection categories. In this study, tomatoes were classified into three maturity stages, i.e., N = 3.

In terms of evaluating efficiency and speed, the computational complexity of the models was determined by analyzing the GFLOPs, parameters, and model size. Meanwhile, inference speed was evaluated using the FPS metric.

## 3. Results

### 3.1. Performance of the Data Augmentation Method

**Figure 12:**
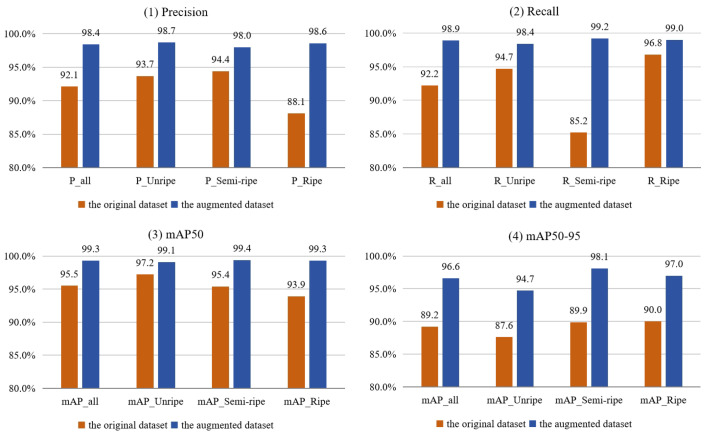
Performance of YOLOv8n models trained with the augmented training dataset and original dataset for detecting tomatoes at different ripening stages.

The effectiveness of the data augmentation methods applied in this study was validated by training the YOLOv8n model on both the augmented and original datasets. The performance of the two models was tested using the 433 images from the test dataset, with the results illustrated in Figure 12. Compared to the YOLOv8n model trained with the original dataset, the YOLOv8n model trained using the data augmentation method adopted in this study showed significantly improved performance in tomato maturity detection.

The test results showed that the overall recognition precision of tomatoes increased by 6.3%, with the precision for three ripeness categories improving by 5.0%, 3.6%, and 10.5%, respectively. In addition, the overall recognition recall increased by 6.7%, with the recall for three ripeness categories rising by 3.7%, 14.0%, and 2.2%, respectively. Furthermore, the overall mAP50 improved by 3.8%, with the mAP50 for three ripeness categories increasing by 1.9%, 4.0%, and 5.4%, respectively. The overall mAP50:95 improved by 7.4%, with the mAP50:95 for three ripeness categories increasing by 7.1%, 8.2%, and 7.0%, respectively. Therefore, the data augmentation method used in this study enhances the detection accuracy of tomato maturity and significantly bolsters the robustness of the model.

### 3.2. Lightweight Module Ablation Experiments

Various lightweight modules were incorporated into YOLOv8n, followed by a series of ablation experiments to evaluate their performance. The proposed network GPC-YOLO was compared with four lightweight networks: MobileNetV4 small [41], GhostNet V2 [42], ShuffleNet V2 [43], and RepViT [44]. The hardware setup and parameter configurations were kept constant throughout the experiment. Table 1 lists the experimental results.

The proposed network has shown improvements in terms of precision. The precision of GPC-YOLO was 98.7%, which was 0.3% to 1.6% higher than that of the other four networks. However, its recall was lower than that of YOLOv8n and ShuffleNetV2, and its mAP50 was also lower than that of YOLOv8n. In addition, the FPS of GPC-YOLO was lower than YOLOv8n, MobileNetV4small, and ShuffleNetV2. Nevertheless, considering the high detection precision of GPC-YOLO, the slight decreases in recall, mAP50, and FPS had a negligible impact on its overall performance.

### 3.3. GPC-YOLO Ablation Experiments

This study used YOLOv8n as the baseline and the GSConv, C2f-PC, CCFF, SimAM, and EIoU modules to conduct ablation experiments. Table 2 and Table 3 list the experimental configuration and the results, respectively. The experimental results showed that the GConv module enhances the precision of YOLOv8n from 98.4% to 99.0%, while also decreasing both parameter count and computational complexity. Although the detection precision of the C2f-PC and CCFF modules decreased slightly, the model’s GFLOPs, parameters, and size were significantly reduced. Moreover, integrating SimAM and EIoU lessened the impact of these lightweight optimizations, resulting in increases in precision, recall, and mAP50:95, from 98.5% to 98.7%, 97.7% to 98.4%, and 94.6% to 95.0%, respectively. Compared to the original model, the precision improved by 0.3%, while GFLOPs, parameters, and model size were reduced by 45.1%, 59.9%, and 57.1%, respectively. Given the high detection performance of precision, recall, FPS, and mAP, the slight decreases in recall, FPS, and mAP have a negligible impact on the overall performance of the model.

Based on the above results, the lightweight approach employed in this study effectively lowered the model’s computational complexity while preserving high detection accuracy and speed.

### 3.4. Comparisons with Other YOLO Versions

**Figure 13:**
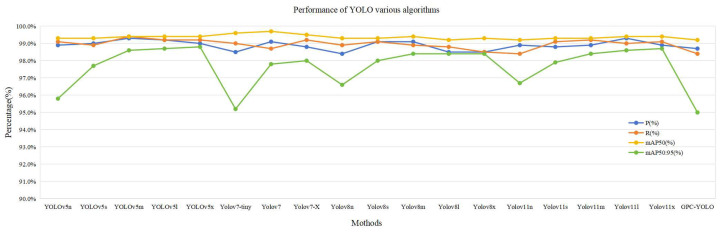
Comparison of the various YOLO algorithms (P, R, mAP50, and mAP50:95).

**Figure 14:**
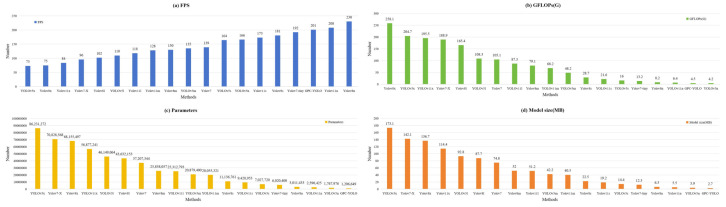
Comparison of various YOLO algorithms (FPS, GFLOPs, Parameters, and Model size).

The superiority of the GPC-YOLO model was examined further by evaluating its performance through a comprehensive comparison with other YOLO versions. Table 4 lists the results. In the three lightweight indicators—GFLOPs, parameters, and model size—the GPC-YOLO model excelled, outperforming all other YOLO versions in terms of parameters and model size. The GFLOPs of GPC-YOLO was only 7.1% higher than YOLOv5n but 45.1% lower than the baseline GFLOPs of YOLOv8n and better than the other YOLO versions, as shown in Figure 13. In terms of the detection rate, the FPS of GPC-YOLO was 201, indicating a high detection rate suitable for deployment on mobile devices. Among all YOLO versions, the precision, recall, mAP50, and mAP50:95 of GPC-YOLO achieved detection accuracies higher than 98.5%, 98.4%, 99.2%, and 95.0%, respectively. GPC-YOLO maintained comparable detection performance to other YOLO versions, as illustrated in Figure 14. The experimental findings indicate that the GPC-YOLO model achieves higher detection performance with lower parameters and computational complexity.

**Figure 15:**
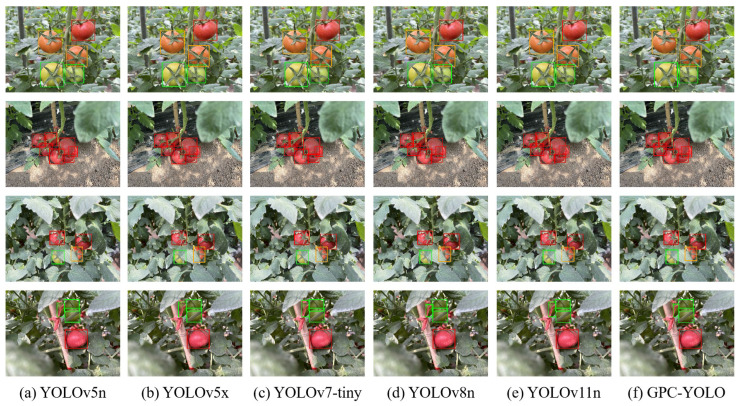
Prediction results of six YOLO versions for tomato fruit and fruit maturity (**a**–**f**).

Finally, multiple scenarios were evaluated to assess the detection efficacy of the GPC-YOLO model within unstructured environments, including tomato overlap, leaf occlusion, branch occlusion, single background, and complex background. The following models were chosen for comparison with the improved GPC-YOLO model developed in this study: the baseline algorithm YOLOv8n, YOLOv5n (which has the lowest GFLOPs), YOLOv5x (which has the highest mAP50:95), YOLOv7-tiny, and YOLOv11n. Figure 15 presents the test results.

The red, orange, and green label boxes indicate the ripe, semi-ripe, and unripe tomatoes, respectively. Experimental results demonstrated that, compared to the other five YOLO models, the GPC-YOLO model achieved comparable detection accuracy in unstructured environments.

### 3.5. Performance Analysis of GPU Memory Utilization

To further evaluate the practicality of GPC-YOLO on resource-constrained devices, experiments were conducted on four hardware platforms: a high-performance GPU (RTX 3090), a low-performance GPU (GTX 1050 Ti), and two edge devices—NVIDIA Jetson AGX Xavier, which serves as a high-performance edge device, and NVIDIA Jetson TX2 NX, which represents a lower-performance edge device. The memory usage and computational performance of GPC-YOLO were compared with the baseline YOLOv8n model in this study. The results demonstrated that GPC-YOLO achieved a better balance between memory consumption and detection performance on resource-constrained devices compared to the original YOLOv8n. The experimental results of GPU memory utilization are shown in Table 5.

The experimental results demonstrate that GPC-YOLO consistently outperforms YOLOv8n in terms of memory efficiency across all tested hardware platforms. On the high-performance GPU (RTX3090), GPC-YOLO allocated 6.8 MB less memory and cached 6 MB less compared to YOLOv8n, resulting in a 15.6% reduction in memory allocation and a 2.8% reduction in cached memory. Although these differences are relatively small on a high-performance GPU, they still highlight GPC-YOLO’s improved memory efficiency. On the low-performance GPU (GTX1050Ti), GPC-YOLO demonstrated a more substantial reduction, utilizing 6.8 MB less memory for allocation and 6 MB less for caching, translating to a 15.6% reduction in memory allocation and a 6.7% reduction in cached memory. This underscores its enhanced efficiency, particularly on lower-end GPUs. The difference becomes even more pronounced on the high-performance edge device, the Jetson AGX Xavier, where GPC-YOLO allocated 6.8 MB less memory and cached 114 MB less than YOLOv8n. This substantial memory saving corresponds to a 58.8% reduction in memory allocation and a 38.8% reduction in cached memory, making GPC-YOLO a more suitable choice for edge computing applications. Finally, the most significant memory savings were observed on the low-performance edge device, the Jetson TX2 NX, where GPC-YOLO utilized 6.8 MB less memory for allocation and 6 MB less for caching compared to YOLOv8n, representing a 58.8% reduction in memory allocation and a 9.1% reduction in cached memory. These results demonstrate GPC-YOLO’s exceptional ability to efficiently operate on resource-constrained devices, highlighting its suitability for low-power edge computing environments.

In summary, the results confirm that GPC-YOLO outperforms YOLOv8n in memory efficiency across all platforms, particularly in resource-limited environments. Its ability to significantly reduce memory allocation and caching while maintaining high detection performance makes it a highly effective solution for deployment on both high-performance and resource-constrained devices.

### 3.6. Discussion

The data augmentation method verification experiment showed that the data augmentation method conducted in this study makes the model more focused on the area of interest within the working range of the robot, significantly improving the detection performance and robustness of the model. Ablation experiments using different lightweight modules combined with YOLOv8n showed that the proposed model was significantly better than other lightweight modules in terms of the lightweight properties while retaining high detection accuracy. The results of the ablation experiments revealed a substantial decrease in the model’s computational complexity and parameter volume following several lightweight module improvements. Although the accuracy was slightly lower than other versions of YOLO models, the P, R, mAP50, and mAP50:95 of the proposed model achieved detection accuracies higher than 98.7%, 98.4%, 99.2%, and 95.0%, respectively. The slight decrease in detection accuracy has a negligible impact on overall model performance. Furthermore, several representative images from unstructured environments in the test set were selected to conduct performance testing. The test results showed that the proposed model could achieve detection comparable accuracy to other YOLO models in different unstructured scenes. Finally, GPC-YOLO demonstrated superior memory efficiency across multiple hardware platforms compared to YOLOv8n. On the RTX 3090, it reduced memory allocation by 15.6% and cached memory by 2.8%. The improvements were more pronounced on the GTX 1050 Ti, with a 15.6% reduction in memory allocation and a 6.7% decrease in cached memory. On edge devices, the Jetson AGX Xavier achieved a 58.8% reduction in memory allocation and a 38.8% decrease in cached memory, while the Jetson TX2 NX showed a remarkable 58.8% reduction in memory allocation and a 9.1% reduction in cached memory. These results highlight GPC-YOLO’s efficiency on resource-constrained devices, making it particularly suitable for low-performance embedded platforms.

In summary, the results suggest that the improvement of the algorithm is lightweight and can effectively identify tomato fruits and fruit maturity in unstructured natural environments. This provides immense potential for tomato ripeness detection and automated picking applications.

## 4. Conclusions

The automated tomato-picking robot relies heavily on object detection technology. Although deep learning detection algorithms are known for their high accuracy, their computational complexity makes them impractical for mobile devices with restricted memory and processing capabilities. In addition, according to natural laws, tomatoes in the same bunch do not mature simultaneously. If the ripe tomatoes are not picked in time, it will affect the development of the immature tomatoes and cause the ripe tomatoes to rot over time. Therefore, in response to the above challenges, this study proposed a lightweight GPC-YOLO tomato-fruit-maturity detection model. The proposed model achieved high detection accuracy for tomatoes and fruit maturity and was sufficiently lightweight. The results showed that, when trained with an enhanced dataset, the precision, recall, mAP50, and mAP50:95 of the model were improved by 13.2%, 9.3%, 5.8%, and 12.6%, respectively. The proposed model outperformed the other lightweight modules and YOLO versions in terms of the parameter count and model complexity. The FLOPs, parameters, and model size were reduced to 4.5 G, 1.2 M, and 2.7 M, with reductions of 45.1%, 59.9%, and 57.1%, respectively. Critically, GPC-YOLO exhibited exceptional memory efficiency across diverse hardware platforms. For low-performance edge devices (Jetson TX2 NX), memory allocation decreased by 58.8% and cached memory by 9.1%. These optimizations highlight its suitability for deployment on resource-constrained devices, particularly in edge computing environments. Furthermore, its high detection accuracy and speed, with mAP50:95 reaching 95.0% and the detection speed reaching 201FPS, which can meet the needs of automated picking, provide valuable references for the real-time operation of picking robots in tomato harvesting.

## Figures and Tables

**Table 1:** Comparison experiments with lightweight models.

Model	P (%)	R (%)	mAP50 (%)	FPS	GFLOPs (G)	Parameters	Model Size (MB)
YOLOv8n	98.4	98.9	99.3	230	8.2	3,011,433	6.3
YOLOv8n+MobileNetV4 small	97.1	96.8	98.9	205	8.0	4,302,073	8.9
YOLOv8n+GhostNet V2	97.8	98.2	99.1	148	8.7	6,334,813	13.3
YOLOv8n+ShuffleNet V2 small	98.4	98.7	99.2	218	7.5	2,793,813	5.9
YOLOv8n+RepViT	98.3	98.4	99.2	186	11.7	4,125,421	8.7
GPC-YOLO	98.7	98.4	99.2	201	4.5	1,206,649	2.7

**Table 2:** Configurations of the ablation experiments.

Experiments	Settings
Ablation	A: GSConv Module
	B: C2f-PC Module
	C: CCFF Module
	D: Simple Attention Mechanism
	E: EIoU loss

**Table 3:** Results of the ablation experiments.

Model	P	R	mAP50	mAP50:95	FPS	GFLOPs	Parameters	Model Size
	**(%)**	**(%)**	**(%)**	**(%)**		**(G)**		**(MB)**
YOLOv8n	98.4	98.9	99.3	96.6	230	8.2	3,011,433	6.3
YOLOv8n+A	99.0	98.4	99.3	96.6	222	7.6	2,731,833	5.7
YOLOv8n+A+B	98.3	98.8	99.2	94.9	228	5.4	1,843,737	3.9
YOLOv8n+A+B+C	98.5	97.7	99.2	94.6	222	4.5	1,206,649	2.7
YOLOv8n+A+B+C+D	98.5	98.2	99.2	95.1	205	4.5	1,206,649	2.7
YOLOv8n+A+B+C+D+E	98.7	98.4	99.2	95.0	201	4.5	1,206,649	2.7
	(+0.3)					(−3.7)	(−1,804,784)	(−3.6)

**Table 4:** Comparison results of YOLO algorithms.

Model	P (%)	R (%)	mAP50 (%)	mAP50:95 (%)	FPS	GFLOPs (G)	Parameters	Model Size (MB)
YOLOv5n	98.9	99.1	99.3	95.8	166	4.2	1,767,976	3.9
YOLOv5s	99.0	98.9	99.3	97.7	164	16.0	7,027,720	14.4
YOLOv5m	99.3	99.4	99.4	98.6	135	48.2	20,879,400	42.2
YOLOv5l	99.2	99.2	99.4	98.7	110	108.3	46,149,064	92.8
YOLOv5x	99.0	99.2	99.4	98.8	73	204.7	86,231,272	173.1
Yolov7-tiny	98.5	99.0	99.6	95.2	192	13.2	6,020,400	12.3
Yolov7	99.1	98.7	99.7	97.8	139	105.1	37,207,344	74.8
Yolov7-X	98.8	99.2	99.5	98.0	96	188.9	70,828,568	142.1
Yolov8n	98.4	98.9	99.3	96.6	230	8.2	3,011,433	6.3
Yolov8s	99.1	99.1	99.3	98.0	181	28.7	11,136,761	22.5
Yolov8m	99.1	98.9	99.4	98.4	130	79.1	25,858,057	52
Yolov8l	98.5	98.8	99.2	98.4	102	165.4	43,632,153	87.7
Yolov8x	98.5	98.5	99.3	98.4	75	258.1	68,155,497	136.7
Yolov11n	98.9	98.4	99.2	96.7	208	6.4	2,590,425	5.5
Yolov11s	98.8	99.1	99.3	97.9	173	21.6	9,428,953	19.2
Yolov11m	98.9	99.2	99.3	98.4	128	68.2	20,055,321	40.5
Yolov11l	99.3	99.0	99.4	98.6	118	87.3	25,312,793	51.2
Yolov11x	98.9	99.1	99.4	98.7	84	195.5	56,877,241	114.4
GPC-YOLO	98.7	98.4	99.2	95.0	201	4.5	1,206,649	2.7

**Table 5:** The experimental results of GPU memory utilization.

Hardware	Model	GPU Memory Allocated (MB)	GPU Memory Cached (MB)
RTX 3090	GPC-YOLO	36.76	212.00
RTX 3090	YOLOv8n	43.56	218.00
GTX 1050 Ti	GPC-YOLO	36.76	84.00
GTX 1050 Ti	YOLOv8n	43.56	90.00
Jetson AGX Xavier	GPC-YOLO	4.76	180.00
Jetson AGX Xavier	YOLOv8n	11.56	294.00
Jetson TX2 NX	GPC-YOLO	4.76	60.00
Jetson TX2 NX	YOLOv8n	11.56	66.00

## Data Availability

The data presented in this study are available on request from the corresponding author.
